# Therapeutic drug monitoring for mood stabilizers in elderly: a case report

**DOI:** 10.1192/j.eurpsy.2025.226

**Published:** 2025-08-26

**Authors:** G. Schoretsanitis

**Affiliations:** Department of Psychiatry, Psychotherapy and Psychosomatics, Hospital of Psychiatry, Zurich, Switzerland

## Abstract

**Abstract:**

The management of elderly patients receiving polypharmacy presents significant clinical challenges due to age-related physiological changes, the prevalence of chronic conditions, and the potential for drug-drug interactions and adverse drug reactions (ADRs). Blood level assessments of medications provide a critical tool for optimizing pharmacotherapy in this population. Here we present the benefits of therapeutic drug monitoring (TDM) and pharmacokinetic assessments in the personalized management of elderly patients with polypharmacy. Regular monitoring of blood drug concentrations can identify drug-drug interactions, related toxicity risks, and adherence issues, thereby informing dosage adjustments to achieve optimal therapeutic outcomes.

**Disclosure of Interest:**

G. Schoretsanitis Consultant of: Dexcel, Saladax, HLS Therapeutics, Speakers bureau of: Saladax, HLS Therapeutics, Lundbeck, Thermo Fisher.

